# Association Between Antimicrobials and Pump Proton Inhibitors Consumption with the Incidence of Nosocomial *Clostridiodes difficile* Infection in High Complexity Hospitals in Costa Rica

**DOI:** 10.3390/antibiotics14040350

**Published:** 2025-03-28

**Authors:** Cristina Fernández-Barrantes, Allan Ramos-Esquivel, Luis Esteban Hernández-Soto, Manuel Ramírez-Cardoce, Luis David Garro-Zamora, José Castro Cordero, Santiago Grau

**Affiliations:** 1 Department of Pharmacology, Faculty of Medicine, Universitat Autònoma de Barcelona, 08193 Barcelona, Spain; cristina.fernandezbarr@autonoma.cat; 2 Department of Pharmacy, Hospital San Juan de Dios, Caja Costarricense de Seguro Social, San José 10103, Costa Rica; 3 School of Medicine, University of Costa Rica, San José 11501, Costa Rica; allan.ramos@ucr.ac.cr; 4 Faculty of Pharmacy, University of Costa Rica, San José 11501, Costa Rica; luis.hernandez@ucr.ac.cr; 5 Infectious Diseases Unit, Hospital San Juan de Dios, Caja Costarricense de Seguro Social, San José 10103, Costa Rica; meramirec@ccss.sa.cr; 6 Department of Pharmacy, Hopital México, Caja Costarricense de Seguro Social, San José 10107, Costa Rica; ldgarro@ccss.sa.cr; 7 Infectious Diseases Unit, Hospital México, Caja Costarricense de Seguro Social, San José 10107, Costa Rica; jancastro@ccss.sa.cr; 8 Department of Pharmacy, Hospital del Mar, Parc de Salut Mar, 08003 Barcelona, Spain

**Keywords:** *Clostridiodes difficile* infection, antimicrobial consumption, pump proton inhibitors consumption, defined daily doses, antibiotic prescription

## Abstract

**Background**: Exposure to antimicrobials and Proton Pump Inhibitors (PPIs) are modifiable risk factors for nosocomial *Clostridiodes difficile* infection (CDI). We investigated the association between these agents and nosocomial CDI over five years. **Methods**: Nosocomial CDI from January 2017 to December 2021 were included. Consumption trends were analyzed using a simple linear regression model. A correlation analysis was performed using Spearman’s test in two ways: without a time interval and with 1-month interval matching. An interrupted time-series method to evaluate the impact of three key temporal breakpoints on CDI incidence rate was performed using the Poisson regression model. **Results**: A downward trend for cephalexin, ceftriaxone, clindamycin, gentamicin, macrolides, metronidazole, and penicillin sodium was identified. In contrast, an upward trend was recognized for amoxicillin, ceftazidime/avibactam, ertapenem, fluconazole, ketoconazole, levofloxacin, and tigecycline. Among the antimicrobials that showed a positive association between consumption and the incidence of CDI are clindamycin and cephalosporins after immediate consumption. Moreover, macrolides and metronidazole presented a positive correlation, in both immediate and delayed consumption. PPIs consumption did not show changes and was not associated with nosocomial CDI incidence. The interrupted time series analysis showed no changes at the breakpoints selected. **Conclusions**: Consumption of clindamycin, cephalosporins, and macrolides showed positive association with CDI, despite having a downtrend in consumption. Specific events, such as the COVID-19 pandemic and the implementation of ASP, have had no correlation with CDI. Further analysis is required in Latin America to advance our understanding of risk factors associated with CDI.

## 1. Introduction

*Clostridiodes difficile* (*C. difficile*) is a Gram-positive, anaerobic bacillus that produces spores and toxins. It is commonly found in the intestinal tract of both humans and animals, as well as in environmental reservoirs. Over the past decade, the rate and severity of *C. difficile* infection (CDI) has risen globally, making it an increasingly major concern among hospital-acquired infections [1]. According to the United States Center of Disease Control and Prevention (CDC), *C. difficile* is considered an antibiotic resistance “urgent” threat, with nearly half a million cases of CDI per year. Out of these, around 30,000 patients will succumb to the illness, costing an estimated USD 5 billion [2]. In 2004, the hypervirulent *C. difficile* polymerase chain reaction (PCR) ribotype 02/toxinotype III was first detected in Canada, the United States, Europe, and in several other countries [3]. CDI presents a wide variety of symptoms, ranging from asymptomatic patients to mild diarrhea, and in certain cases, life-threatening conditions such as pseudomembranous colitis and toxic megacolon [4].

Acquiring CDI involves various risk factors, including exposure of toxigenic strains, prior use of any antimicrobial agent, duration of antimicrobial therapy, and the use of gastric acid suppressants. Additional contributing factors include low host serum immunoglobulin levels, reduced colonic IgA production, advanced age, and severity of any pre-existing conditions. Risk factors can be divided into two main categories: host risks, as previously mentioned, and environmental risks, such as deficient cleaning measures (poor cleaning of contaminated surfaces and hand hygiene methods after contact with patients) and proximity to symptomatic patients, among others [5]. Patients are exposed to the bacteria’s spores through contact with the hands of health personnel, or through the hospital’s environment. In patients with risk factors or presenting a disrupted gut microbiota, *C. difficile* can germinate, proliferate, and secrete toxins, which are responsible for the development of the above symptoms [6].

The use of antimicrobials remains the most important modifiable risk factor for CDI. Ampicillin, amoxicillin, cephalosporins, clindamycin, and fluoroquinolones are the antibiotics that are most often associated with the disease, which also happen to be classified as high-risk antibiotics. However, almost all antimicrobials have been associated with CDI at a molecular, patient, and population level [7]. Despite their prevalence, the degree to which various antimicrobial classes associate with the risk of CDI is not fully understood and could potentially be of interest when making effective stewardship recommendations [8]. Evidence suggests that 60% of inpatients receiving antibiotics are, on average, more likely to acquire CDI, with risk increasing over time with augmented prescription of antimicrobials [9]. Furthermore, a decrease in antimicrobial consumption, due to antimicrobial stewardship interventions that prioritize the limitation of high-risk antibiotics, resulted in a lower incidence of CDI at a hospital level [10].

Concerns have been raised about the potential long-term effects of Proton pump inhibitors (PPIs). There is evidence of their role in predisposition in both *C. difficile* colonization and infection. It is often theorized that a reduction of gastric acid secretion increases the risk of gut microbiota composition; however, the verdict remains ambiguous on this topic [11].

On March 2020, the World Health Organization (WHO) declared COVID-19 a global pandemic, posing a significant challenge to health systems worldwide, affecting not only their ability to respond to the new disease but also their capacity to maintain essential health services for the general population. Among these challenges, hospitals faced a rise in healthcare-associated infections, with CDI being particularly significant because of its impact on treatment outcomes for patients with COVID-19 and CDI coinfections [12,13].

The gastrointestinal tract (GI) is susceptible to COVID-19 infection and can serve as an alternative route for viral transmission and pathogenesis. GI manifestations of COVID-19 are linked to severe disease outcomes and increased mortality across all age groups, particularly among elderly patients. In hospitalized COVID-19 patients, the use of empiric antibiotics for microbial infections, along with experimental antiviral and immunomodulatory treatments, may elevate the risk of antibiotic-associated diarrhea and CDI [14].

Most of the scientific evidence originates from first world regions such as the United States and Europe. This leads to a region-wide pause in published data in less economically developed regions, such as Latin America, thus causing gaps in available knowledge for treating physicians. Available studies from Latin America have focused predominantly on CDI incidence rates, and limited data are available on risk factors associated with the ailment or its consequences (such as mortality and healthcare utilization) [15].

Globally, the CDI incidence rate rages from 1.1 to 631.8 per 100,000 population per year, [16], while in the European Union in the year 2020, the mean incidence of healthcare-associated CDI was 25.8 cases per 100,000 patients [17] and in the United State, CDI incidence rates in 2021 were 110 cases per 100,000 [18]. In Latin America, a study conducted in Brazil in 2021 found a CDI incidence rate of 92 cases per 100,000 patients; however, there is a lack of information on CDI incidence in Central America and the Caribbean [19].

The incidence of CDI is an important outcome, often evaluated following antimicrobial stewardship program (ASP) interventions, due to the high risk of CDI attributed to broad spectrum antibiotic therapy [20]. However, there are differences between *C. difficile* strains and their antimicrobial susceptibility depending on the region or country.

Since 2003, the rise in hypervirulent strains such as NAP1 and NAP9 has coincided with an increase in CDI incidence, severity, complications, recurrence, and mortality. NAP1 strains are characterized by enhanced sporulation and greater resistance to antimicrobials, particularly fluoroquinolones. Meanwhile, NAP9 strains produce the TcdB variant, which is capable of inducing a distinct cytopathic effect [21]. A *C. difficile* outbreak in a major Costa Rican hospital in 2009 was primarily driven by the hypervirulent NAP1 strain (45%). This strain demonstrate resistance to ciprofloxacin, moxifloxacin, and levofloxacin. Although NAP9 and other classical nosocomial strains were also identified, they were present in smaller proportions. Since this outbreak, the distribution of *C. difficile* genotypes in other Costa Rican hospitals has not been documented [22].

The role of antimicrobial consumption is usually well described at patient level; however, there is no conclusive evidence on risk factors at a ward-wide level. As a result, analysis was conducted throughout this ecological study, examining whether the evolution in nosocomial CDI rates in Tertiary Care Hospitals in Costa Rica could have any relationship with antimicrobial and PPIs consumption, expressed in defined daily doses (DDD)/100 bed days. Two different analysis methods were used. First, a correlation test during a five-year series without a time interval and with 1-month interval was conducted that was then followed by the second method, which uses time series analysis at different breakpoints of interest, including after ASP implementation and during the COVID-19 pandemic.

## 2. Results

### 2.1. Incidence of Clostridiodes difficile Infection

**Figure 1:**
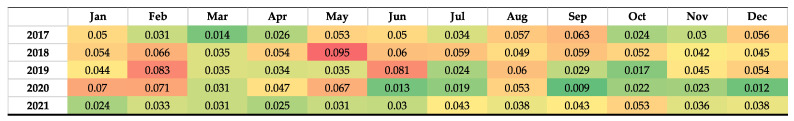
Heatmap showing the incidence of nosocomial *Clostridiodes difficile* infection in Tertiary Care Hospitals in Costa Rica from January 2017 to December 2021, expressed in cases per 100 bed-days. Colors indicate the intensity of data, with warmer colors representing higher values and cooler colors representing lower values.

Between January 2017 and December 2021, a total of 701 nosocomial CDI and 1,636,688 bed-days were identified, proceeded by monthly CDI rate measurement. During the study period, the incidence of nosocomial CDI was lower in the pandemic years (2020 and 2021), reporting a mean of 0.44 infections per 100 bed days and 0.42 infections per 100 bed days, respectively. The lowest infection rate was found in September 2020 with 0.009 CDI per 100 bed days, while the highest rate was reported in 2018 with a total 0.67 infections per 100 bed days. The month with the highest rate was May 2018, reporting 0.095 per 100 bed days. The mean incidence of CDI during this time series was 0.043 infections per 100 bed days. Nosocomial CDI trend per period shows no statistically significant changes; hospital onset CDI in January 2019 is 0.022 (−13.25 to 13.29) *p* = 0.99, in March 2020 is 0.30 (−14.98 to 14.30) *p* = 0.96, and in September 2020 is −1.56 (24.28 to 21.15) *p* = 0.89. Figure 1 shows the evolution of rates of nosocomial CDI for the study cohort.

### 2.2. Antimicrobial Consumption

**Figure 2:**
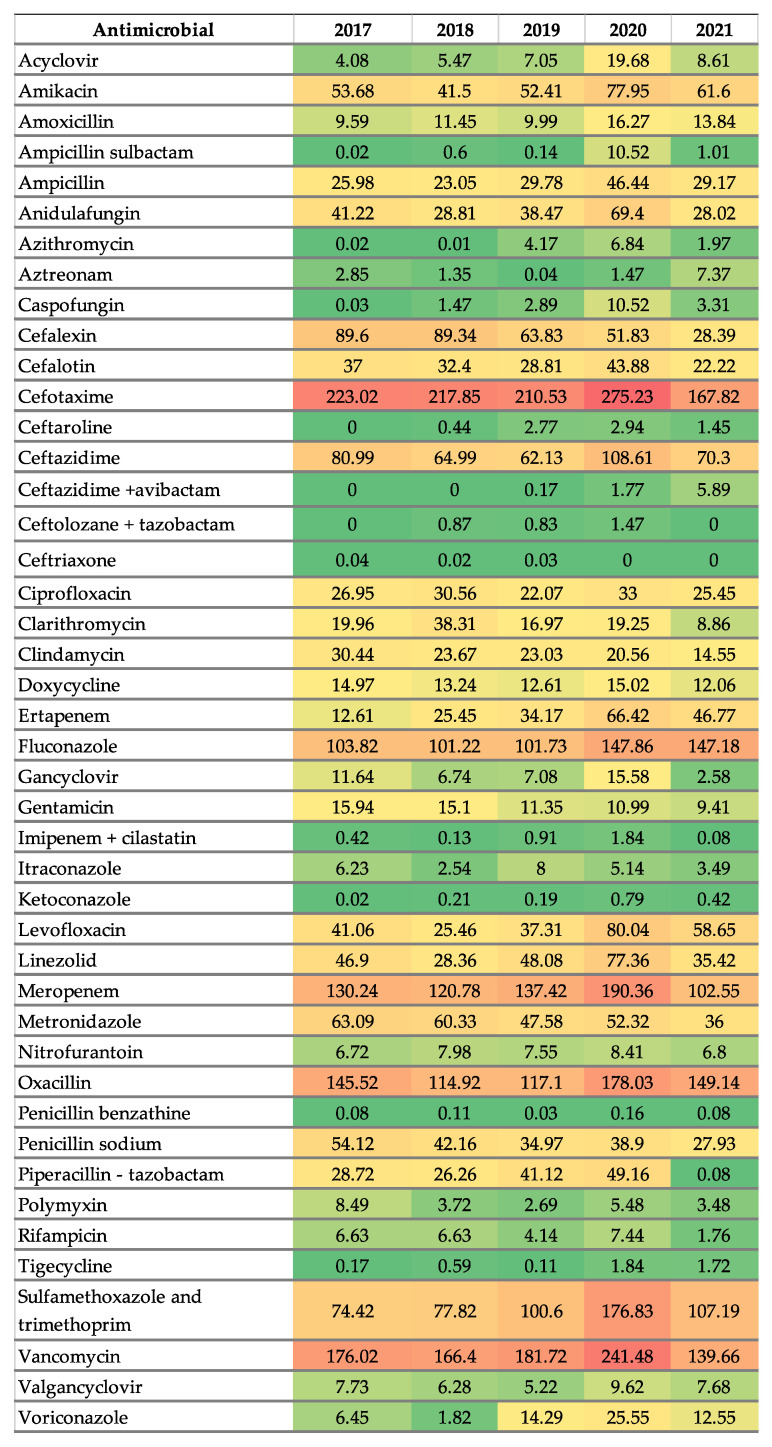
Heatmap of antimicrobial use at participating hospitals from 2017 to 2021, consumption is expressed in DDD/100 bed-day. Colors indicate the intensity of data, with warmer colors representing higher values and cooler colors representing lower values DDD: Defined daily doses.

A total of 1,806,292 DDDs of antibiotics, including 892,616 DDDs of high-risk antibiotics (penicillins, cephalosporins, fluoroquinolones, carbapenems, and clindamycin), were distributed during the five-year series. Figure 2 shows antibiotic consumption trends from 2017 to 2021 in certain selected groups. Antimicrobial consumption trends of most important groups calculated in DDD/100 bed days and DDD/100 discharges from participating hospitals in this same study period were previously published [23]. When analyzing consumption per antibiotic group, cephalosporins were presented as the most used antibiotic group, followed by penicillins and carbapenems.

Nevertheless, the analysis of antibiotic consumption using the World Health Organization Access, Watch and Reserve (WHO AWaRe) classification shows no statistically significant changes (results are presented in Table 1). Consequentially, a more specific statistical analysis was performed, which included individual antimicrobial consumption trends combined with PPIs consumption.

A significant decrease consumption trend in terms of DDD/100 bed days was reported as a group for Macrolides −14.3% AAPC (average annual percent change) 2017–2021, trend −6.54 (*p* = 0.005) and as individual antibiotics for: Cephalexin −0.23% AAPC 2017–2021, trend −15.99 (*p* < 0.0001), Ceftriaxone −0.25% AAPC 2017–2021, trend −0.010 (*p* < 0.0001), Clindamycin −0.16% AAPC 2017–2021, trend −3.48 (*p* < 0.0001), Gentamicin −0.12% AAPC 2017–2021, trend −1.71 (*p* < 0.0001), Metronidazole −0.12% AAPC 2017–2022, trend −6.21 (*p* < 0.0001), and Penicillin sodium −0.14% AAPC 2017–2021, trend −5.56 (*p* < 0.0001).

In contrast, a significant upward trend was identified for the following antimicrobials: Amoxicillin 0.14% AAPC 2017–2021, trend 1.22 *p* = 0.01, Ceftazidime/avibactam 2.9% AAPC 2017–2021, trend 1.35 *p* = 0.0004, Ertapenem 0.50% AAPC 2017–2021, trend 10.92 *p* = 0.0006, Fluconazole 0.11% AAPC 2017–2021, trend 13.33 (*p* = 0.0003), Ketoconazole 3.02% AAPC 2017–2021, trend 0.13 *p* = 0.01, Levofloxacin 0.24% AAPC 2017–2021, trend 8.97 *p* = 0.04, and Tigecycline 4.33% AAPC 2017–2021, trend 0.43 (*p* = 0.001). Table 2 summarizes the antimicrobial consumption trends recognized in the time series.

**Figure 3:**
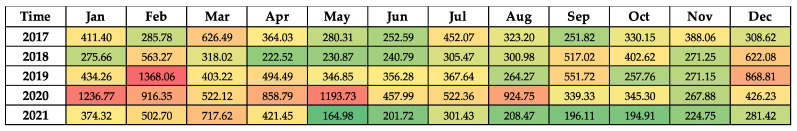
Heatmap of Proton Pump Inhibitors use at participating hospitals in Costa Rica from 2017 to 2021, consumption expressed in DDD/100 bed days. Colors indicate the intensity of data, with warmer colors representing higher values and cooler colors representing lower values. DDD: Defined daily doses.

PPIs do not show statically significant changes during the study period. Figure 3 shows the PPIs consumption trend.

### 2.3. Correlation Between CDI Rates with Antimicrobial Consumption and PPI

Total antimicrobial and PPIs consumption was not significantly associated with nosocomial CDI incidence. Moreover, according to the WHO AWaRe analysis, there was no statistically significant correlation identified.

The following antibiotics showed a positive correlation with a statistically significant downtrend for CDI, in both immediate and delayed consumption: cephalexin (*p* = 0.03 and 0.002), macrolides (*p* = 0.0082 and *p* = 0.0023), metronidazole (*p* = 0.0002 and *p* = 0.01), clindamycin with immediate consumption effect (*p* = 0.0055), and gentamicin with 1-month consumption delayed effect (*p* = 0.02).

In addition, some antibiotics also show positive correlation for CDI, without any statically significant consumption trend. In this scenario, cephalothin describes positive correlation for immediate (*p* = 0.028) and delayed consumption (*p* = 0.0097), and clarithromycin (*p* = 0.0061 and *p* = 0.006) and cephalosporins as a group with immediate consumption (*p* = 0.035).

According to the literature, cephalosporins (such as cephalexin and cephalothin) and clindamycin are considered high-risk antibiotics for CDI development [7,15]. We observed a decrease in the consumption of these antimicrobials (cephalexin and clindamycin). There is a positive correlation for CDI, demonstrating the strong association of these antibiotics as a risk of infection.

On the other hand, antifungals and fluoroquinolones consumption shows a negative statically significant correlation for CDI with 1-month consumption delayed effect (*p* = 0.04 and *p* = 0.0051, respectively) without any changes in antibiotic consumption trends.

Negative statistically significant correlations were also reported for the immediate consumption effect of acyclovir (*p* = 0.01) and voriconazole (*p* = 0.0024). These antimicrobials show independent correlation with consumption trends. Furthermore, ceftazidime/avibactam shows negative correlation with both immediate (*p* = 0.013) and 1-month delayed consumption effect (*p* = 0.002). Finally, levofloxacin shows a negative correlation with immediate effect (*p* = 0.0001). Ceftazidime/avibactam and levofloxacin consumption present a statistically significant upward trend during our time series.

Table 3 presents the complete correlation analysis results for every antimicrobial group and individual antimicrobial without time interval matching and with 1-month interval matching.

### 2.4. Interrupted Time Series Analysis

The correlation analysis of antimicrobial and PPI consumption with the incidence of nosocomial CDI indicates specific positive or negative associations during the five-year time series; however, they do not give information of when these correlations have more impact in the development of nosocomial CDI. For that reason, three interrupted time series analyses were performed using three main breakpoints. The Interrupted time series analysis studied immediate changes in antimicrobial and PPI consumption, as well as trend changes after the breakpoint and their respective relationships with nosocomial CDI rate. There were no statistically significant changes in any of the three breakpoints. Table A1 (Appendix A) summarizes the segmented Poisson’s regression results for this interrupted time series analysis.

## 3. Discussion

To the extent of our knowledge, this article presents the first ecological study in Latin America that combines the evaluation of changes in nosocomial CDI rates coupled with the consumption of antimicrobials and PPIs, while applying correlative tests and interrupted time series analysis using Poisson’s regression model. Throughout the given time frame, no statistically significant changes in nosocomial CDI rates were observed. However, it is noteworthy to point out that there were significant changes in antimicrobial consumption trends, along with the emergence of the COVID-19 pandemic forcing hospitals to adopt infection control measures and strategies to mitigate antimicrobial resistance (e.g., antimicrobial stewardship programs) that were present during the study’s timeframe. The epidemiology of nosocomial *C. difficile* infections has changed over the past years in Europe, North America, and Latin America, with substantial changes in disease incidence and in the prevalence of specific strains like NAP1/027, possibly caused by different burdens of risk factors such as antibiotic use, recent hospitalizations, PPIs use, and increasing age [24,25]

A study published in 2019 that examined the global burden of *C. difficile* estimated that the overall incidence of nosocomial CDI was 4.14 per 10,000 patients-days, ranging from 1.67 (0.58–4.84) in Europe, 2.69 (1.32–5.49) in the Western Pacific, and 6.36 (5.53–7.19) in North America [26].

In this study, the mean incidence of CDI was 0.043 infections per 100 bed days, a lower incidence rate than reported in other regions. As an example, a multicentric study in Germany evaluated nosocomial CDI from 2016 to 2020, reporting 0.30 cases/100 patients in 2016 and 0.26 cases/100 patients in 2020. However, given there are no precise data of the incidence rate in Latin America, no conclusion can be drawn about the regional incidence rate [25,27].

The effect of the COVID-19 pandemic on the incidence of CDI has been explored in several studies, demonstrating mixed findings. While some experts suggested that hospitalized COVID-19 patients might be at higher risk for CDI, most research has reported either no significant change or a decline in CDI cases during the early pandemic years, as shown in our study [28]. Several factors may have influenced this trend, including changes in patient management and the suspension of elective surgeries and routine medical care for non-COVID-19 conditions due to strict lockdown measures. Additionally, enhanced infection prevention strategies, such as visitor restrictions, improved hand hygiene, increased use of personal protective equipment, and the isolation of COVID-19 patients in designed areas, may have contributed to this trend [29].

CDI incidence is a significant outcome often evaluated following AMS program intervention. This is due to the high risk of CDI attributed to broad-spectrum antibiotic use. Risk of CDI conferred varies by agent, with fluoroquinolones, clindamycin, and later-generation cephalosporins associated with higher levels of risk [4,20]. Published research estimates that hospitalized patients receiving antibiotics are on average 60% more likely to acquire CDI. Additionally, prolonged antibiotic exposure, as well as large doses, are correlated with a higher risk of infection. The high prevalence of antibiotic use increases hospital environmental contamination, consequently leading the CDI rate to increase [9].

Brown et al. concluded that, relative to a 7-day course of antibiotics, a 14-day course was associated with 27% more risk, whereas a short course was associated with 9% less risk [30]. Similarly, Owens (2007) concluded that antimicrobials that were formerly thought of as posing low to moderate risk were more commonly associated with CDI thanks to frequent exposure and use [5]. Moreover, the concept of high-risk antibiotics is also explained by Lawes et al. [7], who studied the effect of a national plan to reduce community and hospital antibiotic use associated with CDI. Known as the 4Cs (ciprofloxacin/fluoroquinolones, co-amoxiclav, clindamycin, and cephalosporins), alongside broad-spectrum Beta-lactams, carbapenems and macrolides, these antibiotics were linked with increased risk of CDI at molecular, patient, and population levels. They found that limiting the population use of 4C antibiotics reduces selective pressures and is associated with substantial declines in total CDI [7].

Examining the data obtained in this article, the tendency of antimicrobial consumption changed during this time series analysis. The major findings were an upward trend in amoxicillin, ceftazidime/avibactam, ertapenem, fluconazole, ketoconazole, levofloxacin, and tigecycline. Increased prescription of these compounds could translate to higher antimicrobial exposure in patients. There is further evidence suggesting that ward-level antibiotic exposure is the main risk for CDI, with every 10% increase in ward antibiotic increase being associated with a 1.34-fold increase in *C. difficile* infection risk. A high prevalence of antibiotic use would increase environmental contamination and CDI incidence [9].

It is worthy to point out that this study includes data from the COVID-19 pandemic period, which could influence the statistical significance of antibiotic consumption increases. A study of global antibiotic use during the COVID-19 pandemic (from 2020–2022) found a 10% increase in monthly COVID-19 cases was associated with 0.2–0.3% higher sales of cephalosporins, 0.2–0.3% higher sales of penicillins, 0.4–0.6% higher sales of macrolides, and 0.3% higher sales of all four antibiotics combined per 1000 people. Sales of other antibiotics across continents were also positively associated with COVID-19 cases [31]. Furthermore, in 2020, an outbreak of New Delhi metallo-β-lactamase 1 (NDM) was reported in local hospitals. Consequently, the introduction of ceftazidime/avibactam became mandatory, thus increasing the use of these agents.

This increase in antibiotic use can also be partially explained by the treatment of bacterial coinfection amongst COVD-19 patients, as well as the increased workload for infectious disease professionals. This resulted in the interruption of antibiotic stewardship programs. This initial increase in antibiotic consumption was followed by a reported decrease in antibiotic consumption [32]. In this study, a decrease in cephalexin, ceftriaxone, clindamycin, gentamicin, macrolides, metronidazole, and penicillium sodium was reported. Some of the reasons for the downtrend of these molecules during the study period include a decrease in antibiotic use for respiratory infection, which reflects the effectiveness of personal infection protection efforts, and antimicrobial stewardship interventions as well as people’s hesitancy of visiting hospitalized patients due to the fear of asymptomatic COVID-19 patients, as well as reduced social activities, e.g., city or country lockdown, which decreases the likelihood of acquiring an infection and increased awareness and infection prevention activities among the public (e.g., enhanced hand hygiene, universal wearing of masks, and social distancing) [32].

In this study’s correlation analysis, a positive statistically significant association of CDI with the consumption of gentamicin, cephalosporins (cephalothin, cephalexin), macrolides, clindamycin, and metronidazole were identified. Different classes of medications carry different levels of risk, coupled with the intra-class stratification differences. Included in these differences are compounds such as cephalosporins, clindamycin, penicillin, metronidazole, vancomycin, and fluoroquinolones. It is important to understand that these differences are not fixed but rather evolve according to rapidly changing epidemiology (different strains and different susceptibilities) [3].

Similarly to the findings of our study, the results presented in a study about the global burden and trend of *C. difficile* and its association with world antibiotic consumption in the period 1990–2019 demonstrated a positive association for CDI, with different risk stratification for carbapenems (second highest risk association), cephalosporins, clindamycin (highest risk association), fluoroquinolones, broad-spectrum penicillins, and macrolides; in contrast, trimethoprim-sulfamethoxazole has the lowest risk association [26].

In addition, data from the European Centre for Disease Prevention and Control (ECDC) reported that cephalosporins were the second most used subgroup of antibiotics in hospitals at the European level in 2023. Also, according to this report, carbapenems and third and fourth generation cephalosporins are antibiotics that are the most strongly associated with nosocomial CDI, while a modest association was observed for fluoroquinolones, which is similar to our main finding [12].

According to a study conducted in Colombia, cephalosporins are among the antibiotics more commonly associated with CDI development due to their wide effect on gut microbiota. The risk associated with cephalosporins decreases from third/fourth, to second, to first generation [33]. A strong positive correlation was identified for cephalosporins and clindamycin, even though these antibiotics show a downtrend in consumption. A systematic review and meta-analysis from 2020 concluded that carbapenems and third- and fourth generation cephalosporins remain the most strongly associated with CDI; modest associations were observed for fluoroquinolones, clindamycin, second-generation cephalosporins, and Beta-lactamase inhibitors in combination with penicillin antibiotics [4]. As for gentamicin, there is evidence of a positive correlation between this antibiotic and CDI according to pharmacovigilance reports from the WHO medication monitoring center [34].

A study from Argentina and Mexico concluded that neither cephalosporins nor fluoroquinolones were found in association with CDI [35]. Similarly, in this study’s analysis, fluoroquinolones did not show a positive correlation with CDI. In fact, they showed a statistically significant negative association, which is not dissimilar to that reported for ceftazidime-avibactam, levofloxacin, acyclovir, antifungals, and voriconazole. This could reflect possible bias due to underdiagnosis in certain periods of the COVID-19 pandemic due to changes in healthcare priorities.

This study did not identify statistically significant changes in PPI consumption trends and did not find correlations with CDI incidence, supporting the evidence that suggests a weak association between PPIs use and risk of CDI. In local hospitals, there are protocols and restrictions for PPIs use, which could explain why restrictions on PPIs use could lead to a negative association of these agents with *C. difficile*. However, the literature states that gastric acid suppressants have long been identified as predisposing patients to CDI. Vegetative forms of the bacteria survive on surfaces and could be ingested by patients. Survival of these acid-sensitive vegetative forms in the stomach could be facilitated by two main factors: suppression of gastric acid production by acid-suppressive medications, and presence of bile salts in gastric contents of patients on acid-suppressive therapy. Moreover, PPI use can delay gastric emptying and predispose patients to bacterial overgrowth with associated high intragastric bile salts, which could trigger spore germination in the stomach [36].

Finally, our time series analysis did not show specific changes at predefined breakpoints, such as the beginning of the COVID-19 pandemic and the establishment of ASP programs, meaning that in the present scenarios, these events had no direct relation with CDI incidence.

Although ASP has been associated with a reduction in nosocomial CDI incidence, ASPs that target only specific broad-spectrum antimicrobials can miss opportunities to reduce CDI and antimicrobial resistance in the hospital setting. Also, the overuse of other unnecessary antimicrobials like carbapenems could lead to other nosocomial infections. During the COVID-19 pandemic, ASPs were limited due to other healthcare challenges; consequently, a significant increase in antimicrobial consumption could lead to higher rates of antimicrobial resistance and nosocomial infections like CDI [37].

Several interventions have been implemented worldwide to contain CDI, including guidelines for infection control, education, intensification of hand hygiene promotion, use of additional contact precautions, and improvements in environmental control. Each of these interventions could have played a role in the CDI rate identified in our study. However, investigators suggest that a reduction in the use of high-risk antibiotics is the only apparent driver of the decrease in incidence of nosocomial CDI [24].

These findings suggest that the AMS program has focused its efforts in optimizing cephalosporins and clindamycin consumption as high-risk factors for CDI development, as these antibiotics present a strong relation for CDI development. ASP can help prevent CDI by optimizing antibiotic use, promote infection control measures, and increase public awareness about the disease. One of the main objectives of any ASP should be to make everyone a better steward, including patients and the public in general [38].

This study presents various strengths, key amongst them being the fact that this is the first study in the region that correlates CDI with hospital ward antibiotic prescription and the influence of specific breakpoints, such as Antimicrobial Stewardship Programs and the COVID-19 pandemic. Additionally, the inclusion of antifungals, antivirals, and antibiotics presents a sound base for a broader, more applicable analysis. The limitations of this study lie in the participation of patients for this study. Participation depended on available data from the Tertiary Care Hospitals. Furthermore, it was impossible to account for local antibiotic consumption and specific strain epidemiology like NAP1 or NAP9, yet this could pose a significant impact on nosocomial CDI. In addition, different healthcare priorities during the COVID-19 pandemic and potential diagnostic inconsistencies could have influenced our results. Finally, due to the nature of this ecological study, factors such as comorbidities, age, severity of infection, and the impact of said infection control measures could not be evaluated for analysis. Detailed knowledge about the risk of CDI after antibiotic exposure can aid clinicians in treating high-risk patients, improve antimicrobial stewardship, and, consequently, decrease the incidence of CDI.

Further studies about population level risk factors and CDI correlation, as well as studies to establish the impact of antibiotic susceptibility on the incidence of *C. difficile*, are needed in Latin America.

## 4. Materials and Methods

### 4.1. Study Design and Population

The ecological study conducted involved the examination of antimicrobial and PPIs consumption, correlated with nosocomial DCI incidence, coupled with time-series analysis. Patients with nosocomial CDI admitted to participating General Tertiary Care Hospitals from the Costa Rican Social Security (Hospital San Juan de Dios and Hospital Mexico) between January 2017 and December 2021 were included in the analysis. Participating hospitals are academic centers with the highest complexity level of health attention, covering 85% of the total Costa Rican population. Hospitals were selected based on data availability for antimicrobial and PPIs use, nosocomial CDI rates, and presence of AMS programs. Excluded from the data collection process were pediatric hospital wards, as well as any units that do not generate high discharge rates or occupied bed days (e.g., emergency services). The use of antibiotics in Costa Rican Social Security is regulated by an institutional medicines policy, based on the World Health Organization List of Essential Medicines. This study was approved by the Costa Rican Social Security Ethics Committee: CEC-CENTRAL-CCSS (Protocol #R022-SABI-00319).

### 4.2. Definitions and Surveillance

Nosocomial CDI was defined according to the Clinical Practice Guidelines for the Management of *Clostridioides difficile* Infection in Adults: 2021 Update by SHEA/IDSA [39] as: diarrhea (three or more unformed or liquid stools in less than 24 h) lasting 24 h or longer without any other cause, combined with a positive assay for toxigenic *C. difficile*, diagnosed via nucleic acid amplification test (NAATs) and enzyme immunoassays (EIA) as well as visualization of pseudo membranes by colonoscopy or histopathological diagnosis. A case was categorized as nosocomial if symptoms appeared 3 days or more after admission and up to 8 weeks after discharge. Definitions, laboratory assay to detect CDI and data collection methods did not change during the study period. The detection and categorization of CDI events were done retrospectively by personnel trained in infection prevention and control. Social Security tertiary care hospitals implemented AMS programs starting in January 2018. These programs include the surveillance of all antibiotic prescriptions, and any follow up of pre surgical antibiotic prophylaxis. ASP team gave prospective audits of antimicrobial prescriptions and provided real-time feedback to prescribing physicians.

### 4.3. Variables

Participating hospitals provided monthly nosocomial CDI rates per 100 bed days, according to the Infection Prevention and Control Units records, where this trend was evaluated. Furthermore, monthly data for 44 different antimicrobials available in the participating hospitals and PPIs consumption, calculated in DDD per 100 bed days, was obtained throughout the 60-month study period (January 2017 to December 2021) aided by the pharmaceutical software “Integrated Pharmacy System” (SIFA) version 3.0.5.3. DDD was chosen as the unit of measurement because is the recommended metric by the WHO, as well as reflecting a combination of dosage and duration of treatment [40]. WHO ATC-DDD 2023 index was employed for all 60 months. Before starting the study, the responsible pharmacists were trained in WHO ATC-DDD calculation to guarantee a homogeneous collection of data. Analysis was conducted individually for all 44 different antimicrobials and PPIs. Groups and families of antibiotics were classified according to the WHO AWaRe (Access, Watch and Reserve) classification.

### 4.4. Statistical Analysis

First, as a method of descriptive analysis, annual rates of nosocomial CDI and annual antimicrobial and PPIs consumption trends were described. Changes in antimicrobial consumption during the study period were evaluated using a linear regression test. The AAPC, with 95 percent confidence intervals, for different antimicrobials and PPIs was incorporated into the analysis to measure the average percentage change in antimicrobial consumption over time, thus combining the year-to-year fluctuations into a single value. The statistical analysis was conducted in two main stages: correlation analysis and interrupted time series analysis using Poisson’s regression model. Correlation analysis was performed using Spearman’s test to assess the monotonic association between the monthly antimicrobial and PPIs consumption with CDI incidence rate. This non-parametric test was chosen due to its robustness to non-normal distributions and potential non-linear relationships. The incidence of CDI and the antimicrobial and PPI consumption were paired without time intervals and with 1-month interval to determine the immediate and delayed effects of drug consumption on the incidence of CDI [41].

The second stage of the analysis included interrupted time-series methods to evaluate the impact of key temporal breakpoints on CDI incidence rate. A segmented Poisson’s regression model was employed, given its appropriateness for analyzing count data with time-dependent changes. The interrupted time-series allows for the evaluation of immediate changes in level, coupled with long-term effects due to trend changes. The time series is divided (interrupted) into pre and post breakpoints [24].

Least square regression lines are fitted to each segment of the time series and together expressed by the following model:

Yt=β0+β1 ∗ time1+β2 ∗ breakepoint1+β3 ∗ timeafterbreakepoint1+et

*Yt* represents any outcome at time point (*t*), which in this case is CDI incidence. Time is a continuous variable, measuring the number of months included in the entire time frame (60 months). *β*0 estimated the baseline level at time point zero (y-axis intercept), *β*1 estimates the baseline trend, *β*2 estimates the level change between the pre and post breakpoint period, and *β*3 estimates the change in trend after the breakpoint [42]. The sum of *β*1 and *β*3 estimates the post-breakpoint slope. The error et represents the variability of the model.

We distinguished three main breakpoints. Firstly, one year after the implementation of the ASP in the participating hospitals (January 2019). This was followed by the second and third periods, obtained during the COVID-19 pandemic, the first of which is set in March 2020, the month when the COVID-19 pandemic was officially declared; followed by September 2020, which is the month with the lowest rate of nosocomial CDI in the included time series. *p* values < 0.05 were considered statistically significant. Statistical analysis was performed using SAS Studio OnDemand for Academics (SAS Institute Inc., Cary, NC, USA) Version: 3.81 (Edition Enterprise).

## 5. Conclusions

The use of high-risk antibiotics, such as clindamycin, cephalosporins, and macrolides, were positively associated with CDI, despite some of these antibiotics showing consumption downtrends during our time series analysis. According to our analysis, specific events, such as the COVID-19 pandemic and the implementation of antimicrobial stewardship (ASP) programs, apparently did not impact CDI incidence. Further studies are needed in Latin America to improve our understanding of the role of individual risk factors and infection control measures with CDI.

## Figures and Tables

**Table 1:** Antimicrobial consumption trends according to WHO AWaRe classification, expressed in DDD/100 bed days and DDD/100 discharges.

Total	2017	2018	2019	2020	2021	% AAPC 2017–2021	AAPC 95% CI	Trend (β1)	*p* Value
ACCESS	621.18	553.66	539.78	748.08	519.41	−4.4	−47.6 to 45.0	−0.91	0.97
WATCH	746.69	723.13	752.68	1079.66	623.95	−4.4	−55.3 to 56.4	11.1	0.81
RESERVE	58.42	35.33	54.68	92.34	55.34	−1.3	−82.8 to 104.8	5.08	0.34

WHO AWaRe: World Health Organization antibiotic classification (access, watch and reserve); DDD: defined daily dose; % AAPC: average annual percent change; 95% CI: 95% confidence intervals; Trend β1: the estimated change in antimicrobial consumption per year.

**Table 2:** Antimicrobial consumption trends from 2017 to 2021, expressed in DDD/100 bed days.

Total	2017	2018	2019	2020	2021	% AAPC 2017–2021	AAPC 95% CI	Trend (β1)	*p* Value
Antibiotics	30.36	27.92	28.67	40.86	25.51	−3.42	−32.78 to 25.94	0.32	0.84
Aminoglycosides	69.62	56.59	63.76	88.93	71.01	3.00	−18.50 to 24.50	3.51	0.24
Amikacin	53.68	41.50	52.41	77.95	61.60	0.08	−0.49 to 0.64	5.22	0.08
Gentamicin	15.94	15.10	11.35	10.99	9.41	−0.12	−0.28 to 0.04	−1.71	**<0.0001**
Cephalosporins	430.66	405.92	369.09	485.73	296.08	−5.80	−27.80 to 16.20	−18.93	0.29
Cephalexin	89.60	89.34	63.83	51.83	28.39	−0.23	−0.53 to 0.07	−15.99	**<0.0001**
Cephalothin	37.00	32.40	28.81	43.88	22.22	−0.05	−0.72 to 0.62	−1.81	0.40
Cefotaxime	223.02	217.85	210.53	275.23	167.82	−0.03	−0.49 to 0.42	−5.30	0.61
Ceftaroline	0.00	0.44	2.77	2.94	1.45	1.21	−3.14 to 5.56	0.53	0.06
Ceftazidime	80.99	64.99	62.13	108.61	70.30	0.04	−0.74 to 0.82	2.22	0.67
Ceftazidime + avibactam	0.00	0.00	0.17	1.77	5.89	2.93	−4.15 to 10.02	1.35	**0.0004**
Ceftolozane + tazobactam	0.00	0.87	0.83	1.47	0.00	−0.07	−1.22 to 1.09	0.059	0.73
Ceftriaxone	0.04	0.02	0.03	0.00	0.00	−0.25	−1.28 to 0.78	−0.010	**<0.0001**
Carbapenems and Monobactams	146.13	147.72	172.54	260.09	156.77	6.60	−21.7 to 35.0	13.36	0.27
Aztreonam	2.85	1.35	0.04	1.47	7.37	9.57	−18.44 to 37.57	0.91	0.18
Ertapenem	12.61	25.45	34.17	66.42	46.77	0.50	−0.47 to 1.48	10.92	**0.0006**
Imipenem + cilastatin	0.42	0.13	0.91	1.84	0.08	1.34	−3.79 to 6.48	0.10	0.61
Meropenem	130.24	120.78	137.42	190.36	102.55	0.00	−0.57 to 0.57	1.41	0.87
Fluoroquinolones	68.01	56.02	59.38	113.04	84.09	13.20	−27.10 to 53.50	8.91	0.08
Ciprofloxacin	26.95	30.56	22.07	33.00	25.45	0.03	−0.54 to 0.60	−0.056	0.96
Levofloxacin	41.06	25.46	37.31	80.04	58.65	0.24	−0.89 to 1.37	8.97	**0.04**
Macrolides	50.42	61.99	44.17	46.65	25.39	−14.30	−40.00 to 11.40	−6.54	**0.005**
Azithromycin	0.02	0.01	4.17	6.84	1.97	103.86	−227.27 to 434.98	1.07	0.11
Clarithromycin	19.96	38.31	16.97	19.25	8.86	−0.01	−1.12 to 1.10	−4.12	0.09
Penicillins	264.03	218.55	233.13	339.47	221.26	0.00	−26.40 to 26.40	3.53	0.80
Amoxicillin	9.59	11.45	9.99	16.27	13.84	0.14	−0.44 to 0.72	1.22	**0.01**
Ampicillin + sulbactam	0.02	0.60	0.14	10.52	1.01	25.37	−31.01 to 81.74	1.19	0.30
Ampicillin	25.98	23.05	29.78	46.44	29.17	0.09	−0.57 to 0.75	2.97	0.17
Oxacillin	145.52	114.92	117.10	178.03	149.14	0.04	−0.49 to 0.57	7.03	0.29
Penicillin benzathine	0.08	0.11	0.03	0.16	0.08	0.87	−2.88 to 4.62	0.0041	0.75
Penicillin sodium	54.12	42.16	34.97	38.90	27.93	−0.14	−0.42 to 0.14	−5.56	**<0.0001**
Piperacillin + tazobactam	28.72	26.26	41.12	49.16	0.08	−0.08	−1.14 to 0.98	−3.43	0.49
Sulfamethoxazole + trimethoprim	74.42	77.82	100.60	176.83	107.19	15.90	−20.30 to 52.10	16.45	0.06
Tetracyclines	15.14	13.83	12.72	16.86	13.78	−0.80	−18.00 to 16.40	0.031	0.94
Doxycycline	14.97	13.24	12.61	15.02	12.06	−0.04	−0.31 to 0.22	−0.40	0.23
Tigecycline	0.17	0.59	0.11	1.84	1.72	4.33	−7.97 to 16.63	0.43	**0.001**
Clindamycin	30.44	23.67	23.03	20.56	14.55	−0.16	−0.35 to 0.03	−3.48	**<0.0001**
Linezolid	46.90	28.18	47.76	76.56	34.61	1.80	−41.70 to 45.30	2.47	0.62
Nitrofurantoin	6.72	7.98	7.55	8.41	6.80	0.01	−0.26 to 0.28	0.058	0.77
Polymyxin	8.49	3.72	2.69	5.48	3.48	−0.04	−1.20 to 1.12	−0.82	0.12
Rifampicin	6.63	6.63	4.14	7.44	1.76	−0.09	−1.14 to 0.97	−0.89	0.09
Vancomycin	176.02	166.40	181.72	241.48	139.66	−1.60	−25.40 to 22.20	0.23	0.98
Antifungals	26.30	22.68	27.60	43.21	32.49	4.31	−28.22 to 36.84	3.29	0.05
Anidulafungin	41.22	28.81	38.47	69.40	28.02	0.06	−0.94 to 1.06	1.41	0.76
Caspofungin	0.03	1.47	2.89	10.52	3.31	12.73	−24.75 to 50.21	1.56	0.08
Fluconazole	103.82	101.22	101.73	147.86	147.18	0.11	−0.26 to 0.48	13.33	**0.0003**
Itraconazole	6.23	2.54	8.00	5.14	3.49	0.22	−1.84 to 2.28	−0.28	0.63
Ketoconazole	0.02	0.21	0.19	0.79	0.42	3.02	−4.32 to 10.37	0.13	**0.01**
Metronidazole	63.09	60.33	47.58	52.32	36.00	−0.12	−0.41 to 0.17	−6.21	**<0.0001**
Voriconazole	6.45	1.82	14.29	25.55	12.55	1.60	−4.06 to 7.27	3.59	0.06
Antivirals	4.69	3.70	3.87	8.98	3.77	−4.27	−74.42 to 65.88	−0.34	0.57
Acyclovir	4.08	5.47	7.05	19.68	8.61	0.46	−1.09 to 2.02	2.32	0.10
Gancyclovir	11.64	6.74	7.08	15.58	2.58	0.00	−1.40 to 1.40	−0.92	0.49
Valgancyclovir	7.73	6.28	5.22	9.62	7.68	0.07	−0.75 to 0.89	0.32	0.47
Proton pump inhibitors	356.21	355.88	498.71	667.63	315.82	0.05	−0.62 to 0.73	23.09	0.56

DDD: defined daily dose; % AAPC: average annual percent change; 95% CI: 95% confidence intervals; Trend β1: the estimated change in antimicrobial consumption per year; statistically significant *p* values are in bold.

**Table 3:** Correlation analysis between antimicrobial and proton pump inhibitors consumption and the incidence of nosocomial *Clostridioides difficile* without a time interval and with a 1-month interval matching.

Class of Antibiotics	DDD Per 100 Bed Days Spearman *p* Without Time Interval Matching	*p* Value	DDD Per 100 Bed Days Spearman *p* with a 1-Month Interval Matching	*p* Value
AWaRe				
Access	0.07	0.57	0.041	0.75
Reserve	−0.062	0.63	−0.109	0.41
Watch	0.089	0.49	−0.026	0.84
Antivirals				
Antivirals	−0.113	0.38	−0.186	0.15
Acyclovir	−0.305	**0.01**	−0.217	0.09
Gancyclovir	0.08	0.54	−0.0151	0.90
Valgancyclovir	−0.89	0.49	−0.210	0.10
Antifungals				
Antifungals	−0.202	0.12	−0.26	**0.04**
Anidulafungin	−0.070	0.59	−0.20	0.11
Caspofungin	−0.073	0.57	−0.07	0.57
Fluconazole	−0.201	0.12	−0.27	0.03
Itraconazole	0.047	0.72	−0.12	0.34
Ketoconazole	0.058	0.65	−0.076	0.56
Voriconazole	−0.38	**0.0024**	−0.216	0.10
Antibiotics				
Antibiotics	0.07	0.56	0.015	0.90
Aminoglycosides	0.021	0.86	−0.15	0.25
Amikacin	−0.070	0.59	−0.22	0.08
Gentamicin	0.203	0.12	0.28	**0.02**
Carbapenems	−0.096	0.46	−0.053	0.68
Aztreonam	−0.22	0.08	−0.116	0.37
Ertapenem	−0.077	0.55	−0.074	0.57
Imipenem Cilastatin	0.179	0.17	0.188	0.15
Meropenem	0.00214	0.98	−0.020	0.87
Cephalosporins	0.27	**0.035**	0.14	0.28
Cephalexin	0.274	**0.03**	0.383	**0.002**
Cephalotin	0.28	**0.028**	0.334	**0.0097**
Cefotaxime	0.17	0.17	−0.0315	0.81
Ceftazidime/Avibactam	−0.318	**0.013**	−0.38	**0.002**
Ceftaroline	−0.122	0.35	−0.17	0.17
Ceftazidime	0.087	0.50	−0.056	0.67
Ceftolozano/Tazobactam	0.169	0.19	−0.092	0.48
Ceftriaxone	0.024	0.85	0.070	0.59
Macrolides	0.338	**0.0082**	0.39	**0.0023**
Azitromicina	−0.064	0.62	−0.037	0.78
Clarithromycin	0.350	**0.0061**	0.35	**0.006**
Penicilinas	0.063	0.63	0.027	0.83
Amoxicillin	0.03	0.80	−0.10	0.44
Ampicillin Sulbactam	−0.15	0.25	0.09	0.49
Ampicillin	−0.050	0.70	−0.24	0.06
Oxacillin	0.028	0.82	−0.116	0.38
Penicillin sodium	0.176	0.17	0.23	0.07
Penicillin benzathine	−0.154	0.23	0.02	0.86
Piperacillin/Tazobactam	0.196	0.13	0.116	0.37
Fluoroquinolones	−0.055	0.67	−0.35	**0.0051**
Ciprofloxacin	0.208	0.10	0.244	0.06
Levofloxacin	−0.200	0.12	−0.47	**0.0001**
Tetracyclines	0.048	0.71	0.015	0.90
Doxycycline	0.058	0.65	0.057	0.66
Tigecycline	−0.179	0.17	−0.19	0.12
Other antibiotics				
Clindamycin	0.354	**0.0055**	0.128	0.33
Linezolid	−0.123	0.35	−0.117	0.37
Metronidazole	0.460	**0.0002**	0.30	**0.01**
Nitrofurantoin	0.024	0.85	−0.033	0.80
Polymyxin	−0.0120	0.92	0.0007	0.99
Rifampicin	0.072	0.58	0.25	0.05
Sulfamethoxazole and Trimethoprim	−0.207	0.11	−0.25	0.05
Vancomycin	0.0704	0.59	0.050	0.70
Proton Pump Inhibitors	−0.139	0.28	−0.14	0.26

DDD: defined daily dose. Statistically significant *p* values are in bold.

## Data Availability

The data presented in this study are available on request from the corresponding author.

## Abbreviations

The following abbreviations are used in this manuscript:

PPIs	Proton Pump Inhibitors
CDI	*Clostridiodes difficile* infection
CDC	The United Stated Center of Disease Control and Prevention
GIPCR	Gastrointestinal tractPolymerase chain reaction
ASP	Antimicrobial Stewardship Program
DDD	Defined Daily Dose
WHO	World Health Organization
AWaRe	Access, Watch and Reserve
AAPC	Average Annual Percent Change
SIFA	Integrated Pharmacy System

## Appendix A

**Table A1:** Change in hospital antimicrobial and proton pump inhibitors consumption around major breakpoints and their relationship with the incidence of nosocomial *C. difficile* in Tertiary Care Hospitals in Costa Rica.

Antimicrobials	January 2019			March 2020			September 2020		
	Hospital Onset CDI per Period	Breakpoint	Change in Trend at Breakpoint	Hospital Onset CDI per Period	Breakpoint	Change in Trend at Breakpoint	Hospital Onset CDI per Period	Breakpoint	Change in Trend at Breakpoint
AWaRe									
Access	0.0005 (−0.0095 to 0.0083) *p* = 0.91	−0.2573 (−6.047 to 5.3939) *p* = 0.91	−0.0353 (−0.3994 to 0.2945) *p* = 0.82	0.0008 (−0.0100 to 0.0089) *p* = 0.86	−0.8528 (−9.6531 to 5.0836) *p* = 0.78	0.0214 (−0.4704 to 0.5136) *p* = 0.78	0.0003 (−0.0097 to 0.0080) *p* = 0.95	−1.04 (−14.6290 to 4.6376) *p* = 0.77	0.0715 (−0.6724 to 1.0190) *p* = 0.82
Reserve	0.0036 (−0.0536 to 0.0363) *p* = 0.85	−0.3486 (−6.3406 to 5.4375) *p* = 0.89	−0.0378 (−0.3930 to 0.2941) *p* = 0.81	0.0050 (−0.0520 to 0.0500) *p* = 0.83	−0.9107 (−9.7930 to 4.8564) *p* = 0.77	0.0267 (−0.4590 to 0.5351) *p* = 0.90	0.0016 (−0.0525 to 0.0382) *p* = 0.93	−1.0131 (−14.6226 to 4.7297) *p* = 0.78	0.0708 (−0.6696 to 1.0243) *p* = 0.83
Watch	0.0003 (−0.0056 to 0.0041) *p* = 0.89	−0.2667 (−6.0762 to 5.3863) *p* = 0.91	−0.0328 (−0.3875 to 0.2964) *p* = 0.83	0.0006 (−0.0058 to 0.0054) *p* = 0.82	−0.9156 (−9.7837 to 4.7265) *p* = 0.77	0.0318 (−0.4546 to 0.5595) *p* = 0.89	0.0003 (−0.0058 to 0.0048) *p* = 0.90	−1.0178 (−14.6046 to 4.6610) *p* = 0.78	0.0779 (−0.6753 to 1.0312) *p* = 0.81
Antivirals									
Antivirals	0.0006 (−0.1114 to 0.0460) *p* = 0.98	−0.2011 (−5.6816 to 5.4144) *p* = 0.93	−0.0334 (−0.3876 to 0.2953) *p* = 0.83	0.0040 (−0.1130 to 0.0880) *p* = 0.92	−0.7728 (−10.1954 to 4.8132) *p* = 0.82	0.0143 (−0.4587 to 0.5467) *p* = 0.94	−0.0013 (−0.1134 to 0.0497) *p* = 0.97	−1.0623 (−14.6450 to 4.8109) *p* = 0.77	0.0697 (−0.6772 to 1.0225) *p* = 0.83
Acyclovir	−0.0023 (−0.3019 to 0.0650) *p* = 0.97	−0.2011 (−5.6816 to 5.4144) *p* = 0.93	−0.0334 (−0.3876 to 0.2953) *p* = 0.83	0.0048 (−0.3019 to 0.1549) *p* = 0.94	−0.7324 (−11.8190 to 4.8138) *p* = 0.83	0.0114 (−0.4600 to 0.6259) *p* = 0.95	0.0008 (−0.3176 to 0.0723) *p* = 0.98	−1.0550 (−14.6492 to 5.2825) *p* = 0.77	0.0677 (−0.6860 to 1.0219) *p* = 0.84
Ganciclovir	0.0096 (−0.1965 to 0.1369) *p* = 0.89	−0.2743 (−6.0194 to 5.4043) *p* = 0.91	−0.0360 (−0.3972 to 0.2945) *p* = 0.82	0.0104 (−0.1973 to 0.1552) *p* = 0.89	−0.7208 (−9.4310 to 4.6557) *p* = 0.80	0.0137 (−0.4527 to 0.4991) *p* = 0.94	0.0067 (−0.1974 to 0.1386) *p* = 0.93	−1.0444 (−14.6218 to 4.6417) *p* = 0.77	0.0715 (−0.6713 to 1.0187) *p* = 0.82
Valganciclovir	−0.0077 (−0.3437 to 0.1892) *p* = 0.95	−0.2069 (−5.4417 to 5.3905) *p* = 0.93	−0.0327 (−0.3883 to 0.2973) *p* = 0.83	−0.0001 (−0.0010 to 0.0007) *p* = 0.75	−0.0243 (−0.0427 to −0.0059) *p* = 0.008	0.0000 (−0.0012 to 0.0013) *p* = 0.93	−0.0050 (−0.3455 to 0.2012) *p* = 0.96	−1.0311 (−14.6376 to 4.6705) *p* = 0.78	0.0649 (−0.6696 to 1.0143) *p* = 0.84
Antifungals									
Antifungals	0.0000 (−0.0244 to 0.0194) *p* = 0.99	−0.1941 (−5.9013 to 5.4732) *p* = 0.93	−0.0332 (−0.3911 to 0.2966) *p* = 0.84	0.0006 (−0.0258 to 0.0217) *p* = 0.96	−0.6977 (−10.0415 to 4.9719) *p* = 0.83	0.0095 (−0.4546 to 0.5455) *p* = 0.96	0.0005 (−0.0261 to 0.0189) *p* = 0.95	−1.1049 (−14.7746 to 5.2328) *p* = 0.77	0.0747 (−0.6901 to 1.0552) *p* = 0.83
Anidulafungin	0.0024 (0.0660 to 0.0399) *p* = 0.92	−0.2628 (−6.0873 to 5.4242) *p* = 91	−0.0342 (−0.3878 to 0.2951) *p* = 0.82	0.0047 (−0.065 to 0.0610) *p* = 0.87	−0.8567 (−9.9297 to 4.7077) *p* = 0.79	0.0231 (−0.4529 to 0.5497) *p* = 0.92	0.0023 (−0.0658 to 0.0431) *p* = 0.92	−1.0605 (−14.6330 to 4.6767) *p* = 0.77	0.0753 (−0.6761 to 1.0301) *p* = 0.82
Caspofungin	0.0128 (−0.4322 to 0.2290) *p* = 0.92	−0.2431 (−5.9853 to 5.4011) *p* = 0.92	−0.0325 (−0.3867 to 0.2993) *p* = 0.83	0.0354 (−0.4600 to 0.3618) *p* = 0.84	−0.9395 (−9.7380 to 5.1209) *p* = 0.77	0.0322 (−0.4875 to 0.5582) *p* = 0.89	0.0090 (−0.4552 to 0.2759) *p* = 0.95	−1.0202 (−14.6645 to 4.6453) *p* = 0.78	0.0708 (−0.6870 to 1.0178) *p* = 0.83
Fluconazole	−0.0009 (−0.0366 to 0.0263) *p* = 0.95	−0.1885 (−5.4794 to 5.4019) *p* = 0.93	−0.0315 (−0.3888 to 0.2982) *p* = 0.84	−0.0004 (−0.0373 to 0.0258) *p* = 0.97	−0.5950 (−9.7314 to 4.6668) *p* = 0.84	0.0039 (−0.4515 to 0.5104) *p* = 0.98	0.0005 (−0.0390 to 0.0256) *p* = 0.97	−1.1039 (−15.0177 to 5.3354) *p* = 0.78	0.0714 (−0.6781 to 1.0636) *p* = 0.84
Itraconazole	0.0054 (−0.3994 to 0.01881) *p* = 0.96	−0.2359 (−5.8501 to 5.5260) *p* = 0.92	−0.0338 (−0.3870 to 0.2952) *p* = 0.83	−0.0015 (−0.3965 to 0.1625) *p* = 0.98	−0.6241 (−9.3305 to 4.5433) *p* = 0.82	0.0047 (−0.4503 to 0.4819) *p* = 0.98	0.0037 (−0.3926 to 0.1705) *p* = 0.97	−1.0524 (−14.6372 to 4.6629) *p* = 0.77	0.0670 (−0.6702 to 1.0140) *p* = 0.83
Ketoconazole	0.0467 (−3.1821 to 1.4258) *p* = 0.96	−0.1981 (−5.4691 to 5.3960) *p* = 0.93	−0.0332 (−0.3886 to 0.2957) *p* = 0.83	0.1021 (−3.1558 to 1.8541) *p* = 0.91	−0.6988 (−9.4203 to 4.6571) *p* = 0.81	0.0097 (−0.4637 to 0.4878 *p* = 0.96	0.0561 (−3.1593 to 1.5261) *p* = 0.95	−1.0519 (−14.6398 to 4.6383) *p* = 0.77	0.0683 (−0.6694 to 1.0169) *p* = 0.83
Voriconazole	−0.0024 (−0.1727 to 0.0921) *p* = 0.89	−0.1538 (−6.1370 to 5.6403) *p* = 0.95	−0.0325 (−0.3878 to 0.2968) *p* = 0.83	−0.0002 (−0.1794 to 0.1184) *p* = 0.99	−0.6175 (−9.4550 to 5.4526) *p* = 0.84	0.0045 (−0.5070 to 0.4995) *p* = 0.98	−0.0052 (−0.1707 to 0.0936) *p* = 0.92	−1.0608 (−14.8026 to 4.7114) *p* = 0.77	0.0594 (−0.6986 to 1.0148) *p* = 0.85
Antibiotics									
Antibiotics	0.0002 (−0.0034 to 0.0027) *p* = 0.89	−0.2759 (−6.1584 to 5.3900) *p* = 0.91	−0.0341 (−0.3911 to 0.2951) *p* = 0.83	0.0004 (−0.0036 to 0.0035) *p* = 0.82	−0.9326 (−9.8016 to 4.8605) *p* = 0.77	0.0309 (−0.4599 to 0.5469) *p* = 0.89	0.0002 (−0.0035 to 0.0029) *p* = 0.91	−1.0244 (−14.6110 to 4.6520) *p* = 0.78	0.0759 (−0.6741 to 1.0271) *p* = 0.82
Aminoglycosides	0.0030 (−0.0153 to 0.0305) *p* = 0.87	−0.2689 (−5.8877 to 5.3796) *p* = 0.91	−0.0376 (−0.4007 to 0.2946) *p* = 0.81	0.0046 (−0.0571 to 0.0477) *p* = 84	−0.8860 (−10.0507 to 4.7371) *p* = 0.78	0.0196 (−0.4505 to 0.5315) *p* = 0.92	0.0016 (−0.0569 to 0.0326) *p* = 0.93	−1.0418 (−14.6712 to 4.6642) *p* = 0.77	0.0701 (−0.6691 to 1.0328) *p* = 0.83
Amikacin	0.0029 (−0.0572 to 0.0312) *p* = 0.88	−0.2676 (−5.9186 to 5.3816) *p* = 0.91	−0.0376 (−0.4007 to 0.2946) *p* = 0.81	0.0046 (−0.0571 to 0.0477) *p* = 0.84	−0.8860 (−10.0507 to 4.7371) *p* = 0.78	0.0196 (−0.4505 to 0.5315) *p* = 0.92	0.0016 (−0.0569 to 0.0326) *p* = 0.93	−1.0418 (−14.6712 to 4.6642) *p* = 0.77	0.0701 (−0.6691 to 1.0328) *p* = 0.83
Gentamicin	0.0075 (−0.2371 to 0.1844) *p* = 0.94	−0.1954 (−5.4266 to 5.3899) *p* = 0.93	−0.0333 (−0.3861 to 0.2952) *p* = 0.83	0.0067 (−0.2371 to 0.1840) *p* = 0.94	−0.6146 (−9.3075 to 4.5515) *p* = 0.83	0.0042 (−0.4530 to 0.4816) *p* = 0.98	0.0091 (−0.2366 to 0.1872) *p* = 0.92	−1.0626 (−14.6321 to 4.6383) *p* = 0.77	0.0664 (−0.6688 to 1.0128) *p* = 0.83
Carbapenems	0.0009 (−0.0216 to 0.0139) *p* = 0.91	−0.2563 (−6.0278 to 5.3985) *p* = 0.91	−0.033 (−0.3885 to 0.2970) *p* = 0.83	0.0021 (−0.0228 to 0.0227) *p* = 0.83	−0.9612 (−10.0725 to 4.7836) *p* = 0.77	0.0331 (−0.4581 to 0.5886) *p* = 0.89	0.0009 (−0.0226 to 0.0164) *p* = 0.91	−1.0445 (−14.6088 to 4.6325) *p* = 0.71	0.0786 (−0.6772 to 1.0280) *p* = 0.81
Aztreonam	0.0070 (−0.5594 to 0.3352) *p* = 0.97	−0.1792 (−5.5257 to 5.4647) *p* = 0.94	−0.0346 (−0.4447 to 0.3229) *p* = 0.84	0.0023 (−0.5844 to 0.3442) *p* = 0.99	−0.6225 (−9.3228 to 4.5459) *p* = 0.82	0.0036 (−0.5077 to 0.5251) *p* = 0.98	−0.0015 (−0.6073 to 0.3836) *p* = 0.99	−1.0427 (−14.7872 to 4.7854) *p* = 0.78	0.0671 (−0.6886 to 1.0260) *p* = 0.84
Ertapenem	0.0038 (−0.0700 to 0.0413) *p* = 0.88	−0.2265 (−5.6727 to 5.3886) *p* = 0.92	−0.0311 (−0.3864 to 0.3051) *p* = 0.84	0.0082 (−0.0701 to 0.0716) *p* = 0.79	−0.9865 (−10.1565 to 4.6538) *p* = 0.76	0.0323 (−0.4541 to 0.5656) *p* = 0.88	0.0030 (−0.0714 to 0.0495) *p* = 0.91	−0.9810 (−14.6279 to 5.0792) *p* = 0.79	0.0716 (−0.6685 to 1.0404) *p* = 0.82
Imipenem Cilastatin	0.0258 (−1.1997 to 0.3109) *p* = 0.91	−0.2397 (−5.7173 to 5.3882) *p* = 0.92	−0.0329 (−0.3866 to 0.2970) *p* = 0.83	0.0444 (−1.1975 to 0.5158) *p* = 0.87	−0.7682 (−9.5684 to 4.6221) *p* = 0.80	0.0164 (−0.4594 to 0.5074) *p* = 0.93	0.0124 (−1.2308 to 0.3410) *p* = 0.96	−1.0225 (−14.6374 to 4.8281) *p* = 0.78	0.0670 (−0.6695 to 1.0153) *p* = 0.83
Meropenem	0.0009 (−0.0256 to 0.0196) *p* = 0.93	−0.2458 (−6.0018 to 5.4393) *p* = 0.92	−0.0332 (−0.3879 to 0.2956) *p* = 0.83	0.0019 (−0.0273 to 0.0271) *p* = 0.88	−0.8254 (−9.7619 to 4.7843) *p* = 0.79	0.0233 (−0.4603 to 0.5497) *p* = 0.92	0.0011 (−0.0274 to 0.0221) *p* = 0.92	−1.0670 (−14.6158 to 4.6710) *p* = 0.77	0.0789 (−0.6875 to 1.0223) *p* = 0.82
Cephalosporins	0.0010 (−0.0134 to 0.0118) *p* = 0.87	−0.2691 (−5.9553 to 5.3721) *p* = 0.91	−0.0328 (−0.3895 to 0.2955) *p* = 0.83	0.0014 (−0.0319 to 0.0126) *p* = 0.82	−0.8180 (−9.5002 to 4.7667) *p* = 0.78	0.0254 (−0.4626 to 0.5167) *p* = 0.90	0.0008 (−0.4626 to 0.5167) *p* = 0.90	−0.9912 (−14.5968 to 4.7190) *p* = 79	0.0748 (−0.6701 to 1.0233) *p* = 82
Cephalexin	0.0007 (−0.0560 to 0.0335) *p* = 0.97	−0.1878 (−5.4345 to 5.4309) *p* = 0.93	−0.0321 (−0.3861 to 0.3065) *p* = 0.84	0.0013 (−0.0530 to 0.0434) *p* = 0.95	−0.6159 (−9.3160 to 4.5364) *p* = 0.82	0.0062 (−0.4606 to 0.4821) *p* = 0.97	0.0006 (−0.0510 to 0.0361) *p* = 0.97	−1.0322 (−14.6544 to 4.9047) *p* = 0.78	0.0659 (−0.6708 to 1.0142) *p* = 0.83
Cephalothin	0.0031 (−0.1079 to 0.0474) *p* = 0.92	−0.2308 (−5.7018 to 5.3955) *p* = 0.92	−0.0336 (−0.3886 to 0.2946) *p* = 0.83	0.0040 (−0.1089 to 0.0676) *p* = 0.91	−0.6909 (−9.3205 to 4.7441) *p* = 0.81	0.0131 (−0.4898 to 0.4908) *p* = 0.95	0.0003 (−0.1140 to 0.0504) *p* = 0.99	−1.0408 (−14.7199 to 4.9238) *p* = 0.78	0.0664 (−0.6700 to 1.0145) *p* = 0.83
Cefotaxime	0.0009 (−0.0198 to 0.0162) *p* = 0.91	−0.2547 (−5.8497 to 5.3967) *p* = 0.91	−0.0323 (−0.3858 to 0.2955) *p* = 0.83	0.0014 (−0.0197 to 0.0177) *p* = 0.87	−0.7515 (−9.5098 to 4.5944) *p* = 0.80	0.0188 (−0.4505 to 0.5188) *p* = 0.93	0.0008 (−0.0197 to 0.0165) *p* = 0.92	−1.0258 (−14.6159 to 4.6675) *p* = 0.78	0.0739 (−0.6724 to 1.0241) *p* = 0.82
Ceftazidime/Avibactam	0.0149 (−0.9332 to 0.4016) *p* = 0.95	−0.1759 (−5.4946 to 5.4344) *p* = 0.94	−0.0364 (−0.4067 to 0.3120) *p* = 0.82	0.0194 (−1.0239 to 0.4358) *p* = 0.94	−0.6469 (−9.4814 to 4.5897) *p* = 0.82	0.0001 (−0.4531 to 0.5139) *p* = 0.99	0.0083 (−1.0845 to 0.5016) *p* = 0.97	−1.0599 (−14.8155 to 4.6740) *p* = 0.72	0.0642 (−0.6878 to 1.0166) *p* = 0.84
Ceftaroline	0.0003 (−0.6986 to 0.1869) *p* = 0.99	−0.1957 (−5.5017 to 5.4136) *p* = 0.93	−0.0332 (−0.3862 to 0.2957) *p* = 0.83	−0.0001 (−0.7155 to 0.2331) *p* = 0.99	−0.6215 (−9.3457 to 4.6171) *p* = 0.82	0.0048 (−0.4815 to 0.4819) *p* = 0.98	−0.0103 (−0.7190 to 0.1975) *p* = 0.94	−1.1012 (−14.7423 to 4.9203) *p* = 0.77	0.0676 (−0.6696 to 1.0160) *p* = 0.83
Ceftazidime	0.0211 (−0.0444 to 0.0426) *p* = 0.84	−0.2729 (−5.8625 to 5.3586) *p* = 0.91	−0.0407 (−0.4105 to 0.2951) *p* = 0.80	0.0050 (−0.0441 to 0.04711) *p* = 0.81	−0.8657 (−9.6879 to 4.6134) *p* = 0.77	0.0166 (−0.4482 to 0.5021) *p* = 0.93	0.0032 (−0.0439 to 0.0427) *p* = 0.87	−1.0732 (−14.6659 to 4.7119) *p* = 0.77	0.0747 (−0.6697 to 1.0276) *p* = 0.81
Ceftolozane/Tazobactam	0.0265 (−1.1830 to 0.3649) *p* = 0.92	−0.2028 (−5.4502 to 5.3868) *p* = 0.93	−0.0298 (−0.3877 to 0.3054) *p* = 0.85	0.0454 (−1.1748 to 0.5019) *p* = 0.87	−0.6897 (−9.3541 to 4.5374) *p* = 0.81	0.0144 (−0.4564 to 0.5001) *p* = 0.94	0.0240 (−1.1725 to 0.4229) *p* = 0.93	−1.0012 (−14.6202 to 4.9676) *p* = 0.78	0.0678 (−0.6689 to 1.0160) *p* = 0.83
Ceftriaxone	−0.5503 (−50.6005 to 10.2708) *p* = 0.95	−0.1941 (−5.4509 to 5.4293) *p* = 0.93	−0.0336 (−0.4004 to 0.2975) *p* = 0.83	−0.5907 (−50.4747 to 10.1571) *p* = 0.94	−0.6290 (−9.3234 to 4.5381) *p* = 0.82	0.0052 (−0.4501 to 0.4824) *p* = 0.97	−0.5627 (−50.6230 to 10.1139) *p* = 0.94	−1.0503 (−14.6426 to 4.6402) *p* = 0.77	0.0668 (−0.6694 to 1.0147) *p* = 0.83
Macrolides	0.0025 (−0.1079 to 0.0630) *p* = 0.94	−0.1506 (−5.4861 to 5.5395) *p* = 0.95	−0.0293 (−0.3901 to 0.3156) *p* = 0.86	0.0059 (−0.0994 to 0.0593) *p* = 0.87	−0.6899 (−9.3056 to 4.6110) *p* = 0.81	0.0137 (−0.4757 to 0.4854) *p* = 0.94	0.0024 (−0.1071 to 0.0589) *p* = 0.94	−0.9884 (−14.6888 to 5.0126) *p* = 79	0.0658 (−0.6695 to 1.0136) *p* = 0.83
Azithromycin	−0.0027 (−0.4889 to 0.1127) *p* = 0.98	−0.1795 (−5.6446 to 5.5019) *p* = 0.94	−0.0335 (−0.3865 to 0.2954) *p* = 0.83	−0.0005 (−0.4987 to 0.1943) *p* = 0.99	−0.6192 (−9.3150 to 4.6934) *p* = 0.83	0.0046 (−0.4864 to 0.4844) *p* = 0.98	−0.0082 (−0.5044 to 0.1202) *p* = 0.94	−1.0944 (−14.7725 to 4.6475) *p* = 0.77	0.0662 (−0.6857 to 1.0172) *p* = 0.83
Clarithromycin	0.0037 (−0.1169 to 0.0762) *p* = 0.93	−0.1074 (−5.5663 to 5.8119) *p* = 0.96	−0.0278 (−0.3897 to 0.3210) *p* = 0.87	0.0063 (−0.1039 to 0.0599) *p* = 0.86	−0.6569 (−9.3037 to 4.5210) *p* = 0.81	0.0104 (−0.4521 to 0.4839) *p* = 0.95	0.0040 (−0.1103 to 0.0598) *p* = 0.92	−0.9751 (−14.6237 to 4.9818) *p* = 0.79	0.0653 (−0.6699 to 1.0125) *p* = 0.83
Penicillins	0.0006 (−0.0162 to 0.0126) *p* = 0.93	−0.2502 (−5.9861 to 5.4333) *p* = 0.91	−0.0351 (−0.3935 to 0.2949) *p* = 0.82	0.0011 (−0.0167 to 0.0155) *p* = 0.88	−0.8206 (−9.7186 to 5.0138) *p* = 0.79	0.0176 (−0.4630 to 0.5114) *p* = 0.93	0.0002 (−0.0164 to 0.0125) *p* = 0.98	−1.0438 (−14.6543 to 4.6551) *p* = 0.77	0.0679 (−0.6701 to 1.0163) *p* = 0.83
Amoxicillin	0.0037 (−0.2388 to 0.2461) *p* = 0.97	−0.1863 (−4.8462 to 4.4736) *p* = 0.93	−0.0332 (−0.3431 to 0.2767) *p* = 0.83	0.0091 (−0.3166 to 0.2183) *p* = 0.94	−0.6600 (−9.3043 to 4.6607) *p* = 0.82	0.0072 (−0.4579 to 0.4826) *p* = 0.97	−0.0017 (−0.3143 to 0.2078) *p* = 0.98	−1.0558 (−14.9566 to 4.6997) *p* = 0.77	0.0666 (−0.6712 to 1.0477) *p* = 0.83
Ampicillin Sulbactam	−0.0025 (−0.4620 to 0.0389) *p* = 0.95	−0.1884 (−5.4420 to 5.4000) *p* = 0.93	−0.0335 (−0.3864 to 0.2948) *p* = 0.83	0.0002 (−0.4574 to 0.0810) *p* = 0.99	−0.6265 (−9.8086 to 4.7564) *p* = 0.83	0.0052 (−0.4822 to 0.4979) *p* = 0.98	−0.0041 (−0.4654 to 0.0420) *p* = 0.93	−1.0840 (−14.6877 to 4.6890) *p* = 0.77	0.0664 (−0.6703 to 1.0143) *p* = 0.83
Ampicillin	0.0041 (−0.1093 to 0.0801) *p* = 0.92	−0.2622 (−6.1881 to 5.4273) *p* = 0.91	−0.0338 (−0.3886 to 0.2953) *p* = 0.83	0.0054 (−0.1098 to 0.0947) *p* = 0.91	−0.7225 (−9.4998 to 4.6340) *p* = 0.81	0.0152 (−0.4531 to 0.5201) *p* = 0.94	0.0026 (−0.1101 to 0.0815) *p* = 0.95	−1.0392 (−14.6312 to 4.6463) *p* = 0.77	0.0711 (−0.6730 to 1.0222) *p* = 0.83
Oxacillin	0.0005 (−0.0245 to 0.01777) *p* = 0.95	−0.2183 (−5.6636 to 5.4175) *p* = 0.92	−0.0353 (−0.3995 to 0.2992) *p* = 0.82	0.0008 (−0.0243 to 0.0196) *p* = 0.93	−0.7108 (−9.7012 to 4.7101) *p* = 0.81	0.0081 (−0.4499 to 0.4958) *p* = 0.96	0.0002 (−0.0242 to 0.0166) *p* = 0.98	−1.0566 (−14.6511 to 4.6507) *p* = 0.77	0.0678 (−0.6704 to 1.0189) *p* = 0.83
Penicillin sodium	0.0012 (−0.0829 to 0.0621) *p* = 0.97	−0.1993 (−5.4415 to 5.3934) *p* = 0.93	−0.0338 (−0.3897 to 0.2952) *p* = 0.83	0.0028 (−0.0822 to 0.0707) *p* = 0.93	−0.6774 (−9.3499 to 4.6593) *p* = 0.81	0.0060 (−0.4513 to 0.4818) *p* = 0.97	−0.0001 (−0.0832 to 0.0577) *p* = 0.99	−1.0477 (−14.7006 to 4.6405) *p* = 0.77	0.0664 (−0.6700 to 1.0225) *p* = 0.83
Penicillin benzathine	−0.4872 (−22.9761 to 7.6030) *p* = 0.94	−0.2071 (−5.4255 to 5.3933) *p* = 0.92	−0.0315 (−0.3889 to 0.2973) *p* = 0.84	−0.1632 (−22.9182 to 10.3866) *p* = 0.98	−0.5970 (−9.4315 to 4.8454) *p* = 0.84	0.0039 (−0.4656 to 0.4841) *p* = 0.98	−0.3589 (−22.3922 to 8.0027) *p* = 0.95	−1.0227 (−14.6305 to 4.8773) *p* = 0.78	0.0639 (−0.6752 to 1.0138) *p* = 0.84
Piperacillin/Tazobactam	0.0042 (−0.0788 to 0.0506) *p* = 0.88	−0.3372 (−6.3895 to 5.4647) *p* = 0.89	−0.0281 (−0.3866 to 0.3119) *p* = 0.86	0.0060 (−0.0821 to 0.0642) *p* = 0.85	−0.7485 (−9.3739 to 4.6351) *p* = 0.79	0.0309 (−0.5079 to 0.5642) *p* = 0.90	0.0025 (−0.0910 to 0.0654) *p* = 0.94	−0.9480 (−14.7637 to 5.2800) *p* = 0.81	0.0721 (−0.6824 to 1.0233) *p* = 0.82
Fluoroquinolones	0.0034 (−0.0490 to 0.0390) *p* = 0.87	−0.2477 (−5.9084 to 5.3761) *p* = 0.91	−0.0398 (−0.4155 to 0.2943) *p* = 0.80	0.0057 (−0.0493 to 0.0541) *p* = 0.81	−0.9987 (−10.1611 to 4.7569) *p* = 0.76	0.0238 (−0.4465 to 0.5338) *p* = 0.91	0.0026 (−0.0490 to 0.0404) *p* = 0.89	−1.0794 (−14.7321 to 4.6991) *p* = 0.77	0.0758 (−0.6692 to 1.0445) *p* = 0.81
Ciprofloxacin	0.0060 (−0.1373 to 0.0595) *p* = 0.88	−0.1623 (−5.4154 to 5.4641) *p* = 0.94	−0.0321 (−0.3885 to 0.2992) *p* = 0.83	0.0098 (−0.1267 to 0.0892) *p* = 0.82	−0.7748 (−9.4248 to 4.5496) *p* = 0.79	0.0130 (−0.4516 to 0.4825) *p* = 0.94	0.0048 (−0.1301 to 0.0657) *p* = 0.90	−0.9960 (−14.6064 to 4.7720) *p* = 0.78	0.0647 (−0.6688 to 1.0122) *p* = 0.84
Levofloxacin	0.0028 (−0.0610 to 0.0428) *p* = 0.90	−0.2540 (−5.9819 to 5.3888) *p* = 0.91	−0.0392 (−0.4158 to 0.2993) *p* = 0.81	0.0040 (−0.0602 to 0.0541) *p* = 0.88	−0.8302 (−10.0717 to 4.7572) *p* = 0.79	0.0150 (−0.4489 to 0.5364) *p* = 0.94	0.0021 (−0.0612 to 0.0433) *p* = 0.92	−1.0954 (−14.7874 to 4.9179) *p* = 0.77	0.0747 (−0.6721 to 1.0527) *p* = 0.82
Tetracyclines	−0.0007 (−0.2322 to 0.1326) *p* = 0.99	−0.1960 (−5.4300 to 5.4178) *p* = 0.93	−0.0333 (−0.3864 to 0.2978) *p* = 0.83	−0.0020 (−0.2359 to 0.1328) *p* = 0.98	−0.6262 (−9.3176 to 4.5610) *p* = 0.82	0.0047 (−0.4505 to 0.4826) *p* = 0.98	0.0055 (−0.2223 to 0.1461) *p* = 0.94	−1.0864 (−14.6932 to 4.7053) *p* = 0.77	0.0695 (−0.6700 to 1.0184) *p* = 0.83
Doxycycline	−0.0034 (−0.2487 to 0.1328) *p* = 0.97	−0.1976 (−5.4292 to 5.4082) *p* = 0.93	−0.0334 (−0.3870 to 0.2976) *p* = 0.83	−0.0037 (−0.2544 to 0.1350) *p* = 0.96	−0.6251 (−9.3182 to 4.5419) *p* = 0.82	0.0046 (−0.4509 to 0.4818) *p* = 0.98	0.0024 (−0.2418 to 0.1495) *p* = 0.97	−1.0615 (−14.6577 to 4.7202) *p* = 0.77	0.0673 (−0.6723 to 1.0148) *p* = 0.83
Tigecycline	0.0230 (−1.0965 to 0.3221) *p* = 0.92	−0.1823 (−5.4245 to 5.3987) *p* = 0.93	−0.0326 (−0.3876 to 0.2947) *p* = 0.83	0.0124 (−1.1002 to 0.3734) *p* = 0.96	−0.6016 (−9.3097 to 4.7612) *p* = 0.83	0.0052 (−0.4506 to 0.4875) *p* = 0.97	0.0279 (−1.0802 to 0.3436) *p* = 0.90	−1.0728 (−14.7052 to 4.6447) *p* = 0.77	0.0705 (−0.6716 to 1.0265) *p* = 0.82
Clindamycin	0.0063 (−0.1528 to 0.1220) *p* = 0.92	−0.2619 (−5.7069 to 5.4470) *p* = 0.91	−0.0369 (−0.4103 to 0.2983) *p* = 0.82	0.0025 (−0.1520 to 0.0981) *p* = 0.96	−0.6220 (−9.3111 to 4.5506) *p* = 0.82	0.0052 (−0.4509 to 0.4818) *p* = 0.97	0.0014 (−0.1530 to 0.0958) *p* = 0.98	−1.0422 (−14.6440 to 4.6435) *p* = 0.77	0.0661 (−0.6706 to 1.0144) *p* = 0.83
Linezolid	0.0031 (−0.0378 to 0.0439) *p* = 0.88	−0.3288 (−5.3332 to 4.6755) *p* = 0.89	−0.0352 (−0.3460 to 0.2756) *p* = 0.82	0.0051 (−0.0585 to 0.0546) *p* = 0.83	−0.9266 (−10.0027 to 4.7608) *p* = 0.77	0.0290 (−0.4552 to 0.5611) *p* = 0.90	0.0014 (−0.0595 to 0.0377) *p* = 0.94	−1.0183 (−14.6307 to 4.7429) *p* = 0.78	0.0708 (−0.6724 to 1.0240) *p* = 0.83
Metronidazole	0.0086 (−0.0837 to 0.0698) *p* = 0.81	−0.1923 (−5.4642 to 5.4194) *p* = 0.93	−0.0288 (−0.3845 to 0.3065) *p* = 0.85	0.0081 (−0.0829 to 0.0762) *p* = 0.82	−0.5768 (−9.2517 to 4.5816) *p* = 0.84	0.0155 (−0.4470 to 0.4857) *p* = 0.95	0.0082 (−0.0838 to 0.0729) *p* = 0.82	−0.9575 (−14.5238 to 4.9156) *p* = 0.79	0.0690 (−0.6719 to 1.0088) *p* = 0.83
Nitrofurantoin	−0.0039 (−0.5718 to 0.3932) *p* = 0.98	−0.1986 (−5.4531 to 5.3944) *p* = 0.93	−0.0339 (−0.4006 to 0.2956) *p* = 0.83	0.0017 (−0.5305 to 0.3833) *p* = 0.99	−0.6220 (−9.3190 to 4.5470) *p* = 0.82	0.0052 (−0.4598 to 0.4828) *p* = 0.97	0.0013 (−0.5313 to 0.3821) *p* = 0.99	−1.0457 (−14.7024 to 4.6427) *p* = 0.77	0.0665 (−0.6754 to 1.0156) *p* = 0.83
Polymyxin B	0.0084 (−0.3206 to 0.1544) *p* = 0.93	−0.2024 (−5.4773 to 5.3986) *p* = 0.93	−0.0349 (−0.3883 to 0.2947) *p* = 0.82	0.0032 (−0.3188 to 0.1621) *p* = 0.97	−0.6172 (−9.3126 to 4.5545) *p* = 0.83	0.0050 (−0.4504 to 0.4834) *p* = 0.97	0.0064 (−0.3186 to 0.1568) *p* = 0.95	−1.0568 (−14.6559 to 4.6370) *p* = 0.77	0.0676 (−0.6704 to 1.0171) *p* = 0.83
Rifampicin	−0.0140 (−0.4889 to 0.2730) *p* = 0.93	−0.2180 (−5.4304 to 5.4267) *p* = 0.92	−0.0356 (−0.3864 to 0.3053) *p* = 0.82	0.0067 (−0.4801 to 0.3678) *p* = 0.97	−0.6649 (−9.5070 to 5.0639) *p* = 0.83	0.0079 (−0.5046 to 0.4958) *p* = 0.97	−0.0157 (−0.4863 to 0.3025) *p* = 0.93	−1.0939 (−14.7116 to 4.6694) *p* = 0.77	0.0630 (−0.6846 to 1.0128) *p* = 0.84
Sulfamethoxazole and Trimethoprim	0.0001 (−0.0382 to 0.0290) *p* = 0.99	−0.1951 (−5.8733 to 5.4199) *p* = 0.93	−0.0332 (−0.3880 to 0.2968) *p* = 0.83	0.0010 (−0.0386 to 0.0384) *p* = 0.95	−0.6968 (−9.6623 to 4.7622) *p* = 0.82	0.0113 (−0.4554 to 0.5422) *p* = 0.96	0.0006 (−0.0386 to 0.0353) *p* = 0.97	−1.0616 (−14.6365 to 4.9949) *p* = 0.77	0.0714 (−0.7402 to 1.0268) *p* = 0.83
Vancomycin	0.0009 (−0.0233 to 0.0157) *p* = 0.91	−0.2509 (−5.9697 to 5.4052) *p* = 0.91	−0.0326 (−0.3859 to 0.2958) *p* = 0.83	0.0015 (−0.0228 to 0.0174) *p* = 0.87	−0.7914 (−9.5916 to 4.7207) *p* = 0.79	0.0218 (−0.4588 to 0.5312) *p* = 0.92	0.0007 (−0.0228 to 0.0166) *p* = 0.93	−1.0279 (−14.6215 to 4.6666) *p* = 0.78	0.0738 (−0.6754 to 1.0275) *p* = 0.82
Proton pump inhibitors	0.0004 (−0.0059 to 0.0050) *p* = 0.86	−0.3358 (−6.2083 to 5.4348) *p* = 0.89	−0.0260 (−0.3920 to 0.3111) *p* = 0.87	0.0005 (−0.0061 to 0.0048) *p* = 0.84	−0.6949 (−9.3610 to 4.5548) *p* = 0.80	0.0263 (−0.4669 to 0.5397) *p* = 0.90	0.0004 (−0.0062 to 0.0048) *p* = 87	−0.9483 (−14.5743 to 5.0486) *p* = 0.80	0.0808 (−0.6678 to 1.0348) *p* = 0.81
